# Impact of Time-Restricted Feeding and Dawn-to-Sunset Fasting on Circadian Rhythm, Obesity, Metabolic Syndrome, and Nonalcoholic Fatty Liver Disease

**DOI:** 10.1155/2017/3932491

**Published:** 2017-11-19

**Authors:** Ayse L. Mindikoglu, Antone R. Opekun, Sood K. Gagan, Sridevi Devaraj

**Affiliations:** ^1^Michael E. DeBakey Department of Surgery, Division of Abdominal Transplantation, Baylor College of Medicine, Houston, TX, USA; ^2^Department of Medicine, Section of Gastroenterology and Hepatology, Baylor College of Medicine, Houston, TX, USA; ^3^Department of Pediatrics, Division of Gastroenterology, Nutrition and Hepatology, Baylor College of Medicine, Houston, TX, USA; ^4^Clinical Chemistry and Point of Care Technology, Texas Children's Hospital and Health Centers, Department of Pathology & Immunology, Baylor College of Medicine, Houston, TX, USA

## Abstract

Obesity now affects millions of people and places them at risk of developing metabolic syndrome, nonalcoholic fatty liver disease (NAFLD), and even hepatocellular carcinoma. This rapidly emerging epidemic has led to a search for cost-effective methods to prevent the metabolic syndrome and NAFLD as well as the progression of NAFLD to cirrhosis and hepatocellular carcinoma. In murine models, time-restricted feeding resets the hepatic circadian clock and enhances transcription of key metabolic regulators of glucose and lipid homeostasis. Studies of the effect of dawn-to-sunset Ramadan fasting, which is akin to time-restricted feeding model, have also identified significant improvement in body mass index, serum lipid profiles, and oxidative stress parameters. Based on the findings of studies conducted on human subjects, dawn-to-sunset fasting has the potential to be a cost-effective intervention for obesity, metabolic syndrome, and NAFLD.

## 1. Global Impact of Obesity, Metabolic Syndrome, Nonalcoholic Fatty Liver Disease, and Hepatocellular Carcinoma

In 2014, the World Health Organization estimated that there were more than 1.9 billion adult overweight people of whom more than 600 million were obese [1]. More than 115 million people are estimated to have obesity-associated diseases (e.g., cardiovascular disease, stroke, diabetes, dyslipidemia, obstructive sleep apnea, metabolic syndrome, and breast, colon, and liver cancers) [1, 2]. A prospective, 16-year study of 900,053 US obese adults showed that subjects with a body mass index (BMI) ≥ 40 kg/m^2^ had 52% to 62% higher death rate from cancers compared to those with normal weight [3]. Obesity and metabolic syndrome are strong risk factors for nonalcoholic fatty liver disease (NAFLD), a chronic liver disease that can result in cirrhosis and liver cancer [4–6]. To compound this crisis, between 1960 and 2010, the obesity in adults in the US increased from 13% to 36% [7], which foretells of increasing significance. Aside from the negative health consequences, like liver disease and diabetes, the socioeconomic impact of these sequelae is profound [8]. The ramifications are far-reaching, and better approaches to addressing NAFLD are needed.

The Third National Health and Nutrition Examination Survey conducted between 1988 and 1994 reported that ~20% of the U.S. population had NAFLD [9]. A subsequent study showed a nearly 3-fold increase in NAFLD prevalence between 2003 and 2011 [10]. It has been projected that over 64 million U.S. residents will be diagnosed with NAFLD and incur about $103 billion annual direct medical costs [11]. While it has been argued that the increase in obesity and its sequelae is a consequence of easy access to caloric dense food and concomitant decrease in physical activity [12], the cofactors responsible for the NAFLD epidemic remain poorly understood. These factors include lipotoxicity (e.g., increased hepatic free fatty acid uptake and de novo lipogenesis, decreased beta-oxidation of free fatty acids), hormonal and cytokine secretion from adipose tissue, genetic and epigenetic factors, mitochondrial dysfunction, endoplasmic reticular stress, several medications, insulin resistance, such that diabetes mellitus is frequently part of the constellation, and unfavorable alterations in gut microbiota (e.g., transformation of lean gut microbiota into obese gut microbiota) that effect deleterious changes to the gut-liver axis [13].

Changes in diet and exercise are the mainstay preventative approaches for NAFLD. However, the majority of obese patients are unable to accomplish or sustain intentional weight reduction by diet and exercise alone [14]. Medical therapies (e.g., drugs) have had limited benefit [15, 16]. For example, Sanyal et al. [17] showed that among subjects with NAFLD without diabetes, only 47% of subjects treated with pioglitazone and 36% of those treated with vitamin E had a resolution of nonalcoholic steatohepatitis (NASH). Although this study showed significant improvement in total NAFLD activity score in patients with NAFLD treated with pioglitazone and vitamin E compared to those who received placebo, there was no significant improvement in the hepatic fibrosis score compared to placebo [17]. In contrast, bariatric surgery has been shown to improve hepatic steatosis, fibrosis, and necroinflammatory activity in the morbidly obese patients with NASH [18, 19] but the mechanisms have not yet been fully worked out. Murine studies on obese animals following Roux-en-Y gastric bypass demonstrated favorable alteration in the abundance of colonic Gammaproteobacteria (*Escherichia*) and Verrucomicrobia (*Akkermansia*) and suggest that gut microbiota indirectly contribute to reduced host weight and recurrent adiposity [20]. Numerous complementary and alternative remedies have been tested and have shown some promise in ameliorating NAFLD, but none have been proven to have significant efficacy [21, 22] and some have been shown to cause drug-induced liver injury [23].

Over the last two decades, human and animal studies have shown that timing of meal intake is as important as the composition of the diet and caloric quantity to prevent obesity and its complications [24–33]. Mealtime and cultural eating habits, the quantity, and type of foods ingested can adversely affect health status and increase the likelihood of developing obesity and related complications. For example, it was recently shown that subjects that were in a low mealtime habits quality group (mealtime habits quality was based on mealtime circumstances including whether there was time for eating, distraction during food intake, and environmental, social, or cultural habits) had significantly higher risk for metabolic syndrome and insulin resistance [34]. Conversely, subjects who were in high mealtime habits quality group were deemed to be at lower risk for adversity [34]. As such, the mechanisms by which mealtime influences adverse outcomes, or conversely, can ameliorate adverse outcomes, such as by modulating circadian rhythm, demand further investigations.

It has been suggested that time-restricted food intake might be a successful intervention to prevent and manage obesity, metabolic syndrome, and its complications due to neurohormonal adaptions [24–33]. Altering feeding time, whether it is a primary intervention or adjunctive to dietary caloric content, may have a net benefit, especially to those most afflicted. Several recent rodent studies have shown that dysregulation of the circadian secretion of melatonin increases the risk for diabetes and sequelae [35–38], and supplemental melatonin administration resulted in a modest improvement in the parameters of human metabolic syndrome [35]. Melatonin release is multifactorial, and favorable melatonin response points toward a complex relationship between mealtime, the fasting state, and neurohormonal factors that invite for further study. As such, the roles of mealtime and sleep-wake cycles appear to be important. Herein, we summarize the data regarding time-restricted feeding and dawn-to-sunset fasting on obesity and point toward potentially innovative strategies to curtail NAFLD.

## 2. Impact of Time-Restricted Feeding on Master and Hepatic Clocks in Murine Models

Micronutrient availability promotes cellular growth and proliferation through various inductive mechanisms, such as amino acid sensing and reactive activation of the mechanistic mammalian target of rapamycin complex 1, the central metabolic regulator [39]. The induction of ribosomal biosynthesis appears to be as crucial and is a highly regulated step in proliferative growth, cancer, and cell survival under stress [40]. Cells which attempt to conserve the ribosomal biosynthetic mechanisms at all costs are thought to promote cell survival and appear important in the malignant process [40]. These mechanisms are modulated, and like NAFLD, a multifactorial premalignant condition [6] appears to be under the influence of multiple circadian processes [41, 42]. The associations between NAFLD, circadian rhythm disturbances, and malignancy are poorly understood. Guerrero-Vargas et al. [43] recently demonstrated that constant light exposure appears to promote tumor growth in rats in a way similar to a high caloric diet. As such, the relationships between dietary content, meal timing, circadian rhythm, NAFLD, and malignancy appear complex and deserve much greater attention.

Centrally, the suprachiasmatic nucleus (SCN) of the hypothalamus hosts a molecular clock that serves as the driving force for circadian rhythmicity [44, 45] and manipulation of circadian rhythm appears to be a therapeutic target [46–48]. The molecular basis for circadian rhythmicity is thought to be related to an autonomous transcription-translational feedback loop system (positive and negative) [44, 45]. This biomechanism is not limited to the brain alone as circadian clocks also exist in the peripheral tissues, such as adipose tissue and liver [44, 45, 49, 50]. It is thought that peripheral circadian clocks, including those in the liver, have an important role in synchronization of peripheral metabolic regulators to feeding/refeeding cycles and energy demands [33, 44, 45, 49–51]. This appears to be achieved, in part, by modulating the expression of transcription factors, signaling molecules, substrate transporters, and metabolic enzymes [33, 44, 45, 49–51].

The hepatic biological clock can be set or reset by two major mechanisms, light and mealtime [44, 45, 52–56]. The first mechanism involves the stimulation of the master clock located in the SCN of the anterior hypothalamus by light-dark cycles, and this appears to be a dominant factor [44, 45, 52–56]. Information on light-dark cycles is received and transferred to the master clock via retinohypothalamic tract/melanopsin pathway [45] and mammalian target of rapamycin signaling pathway [41]; thereby, the master clock synchronizes with the peripheral clocks, including the hepatic clock via several neural and hormonal output mechanisms [41, 45]. Melatonin-binding sites [57] may play a major role in synchronization between central clock and hepatic clock. According to current clock models, communication between central clock and hepatic clock is facilitated by the sympathetic nervous system and/or hormones [58]. In humans, one of the hormones that may be providing the communication between central clock and hepatic clock could be melatonin via melatonin-binding sites. While the sympathetic nervous system may be the predominant mechanism in mice due to extremely low levels of melatonin, circulating melatonin may be the predominant communication mechanism in humans. However, this remains to be determined.

A secondary hepatic clock mechanism functions in response to time-restricted feeding that releases the hepatic clock from the control of the master clock in the SCN [52, 53, 55]. In this way, the hepatic clock is entrained directly by time-restricted feeding cycles, not by light-dark phase cycles and this phenomenon is independent of the master clock located in the SCN [52, 53, 55]. As such, mealtime appears to be critical in circadian rhythm because the two mechanisms appear to be intertwined.

In contrast to humans, most food consumption and activity in mice occur during night time (dark phase) [52–55, 59, 60]. Murine studies showed that time-restricted feeding changed and reset the phase of hepatic clock oscillations without changing the phase of the master circadian clock only on three distinct occasions: no access to food during dark phase (activity phase) (1) with unlimited access to food during 12-hour light phase, (2) with time-restricted access to food during the light phase, and (3) with time-restricted access to food during subjective day (during daytime 6 am to 6 pm or circadian time 5 to 9) of 24-hour constant darkness [52, 53, 55]. Paradoxically, unlimited food access only during dark phase or ad libitum feeding in mice did not result in significant change in the phase of hepatic circadian clock [52–55, 59]. Damiola et al. [52] showed that no access to food during dark phase with unlimited access to food during light phase completely inversed the phase of mRNA expression in the hepatic clock and clock-controlled genes including Per1, Per2, Per3, Cry1, D site-binding protein (Dbp), Rev-erb*α*, and Cyp2a5. In summary, several murine studies showed that time-restricted feeding during light phase under 12-hour light/12-hour dark cycle or during subjective light phase under 24-hour constant darkness for at least several days resets the phase of hepatic clock [52–55, 59, 60] and this occurs independent of central circadian clock in the SCN [52, 53, 55]. The mechanism by which time-restricted feeding resets the hepatic clock independently of the central clock remains to be discovered.

Hatori et al. [33] showed that time-restricted access to food during dark phase (only 8 hours) with no access to food during light phase under 12-hour light/12-hour dark cycle in mice on a high-fat diet resulted in an increase in the peak-to-trough ratio of mRNA expression of hepatic clock genes including Per1, Per2, Bmal1, Rev-erb*α*, Cry1, Clock, Ror*α*, and Dbp when compared to mice on high-fat diet ad libitum. Hara et al. [53] showed that complete food removal during dark phase with time-restricted access to food during light phase (only 4 hours) under 12-hour light/12-hour dark cycle or time-restricted access to food (only 4 hours) during constant darkness in mice gradually increased the intensity of mRNA expression in the hepatic clock genes including mPer1 and mPer2. These genes encode for critical components of the mammalian circadian clock such that mutations impair mammalian circadian oscillations [61]. Collectively, the studies conducted by Hatori et al. [33], Hara et al. [53], and Sherman et al. [59] suggest that in order to improve the amplitude of hepatic clock oscillations, either no access to food during dark phase with time-restricted access to food during light phase, time-restricted access to food during dark phase with no access to food during light phase under 12-hour light/12-hour dark cycle, or time-restricted access to food during 24-hour constant darkness for several consecutive days to several weeks is necessary in murine models. The ramifications of these studies for the human condition are profound.

## 3. Impact of Time-Restricted Feeding on Metabolic Profile, Obesity, NAFLD, and Inflammatory Markers in Murine Models

Results of several murine studies showed that timing to food access, in the fasting state, is the cue “Zeitgeber” to entrain hepatic oscillators [33, 52–55, 59] and thereby evoke upregulation of mRNA and various protein synthetic pathways, including enzymes that play a role in carbohydrate and lipid metabolism [33, 59]. Hatori et al. [33] also demonstrated that mice fed by high-fat diet ad libitum developed obesity while mice fed by high-fat diet only for 8 hours during dark phase under 12-hour light/12-hour dark cycle did not develop obesity. The fasting period was sixteen hours a day for more than hundred days, and both mouse groups consumed equivalent calories [33]. That study showed that the increase in mRNA expression of hepatic clock genes corresponded to favorably altered mRNA expression of key metabolic regulator enzymes of glucose and fatty acid metabolism (e.g., increase mRNA expression of glucose-6-phosphate dehydrogenase and hepatic lipase, decrease mRNA expression of pyruvate carboxylase, glucose-6-phosphatase, and fatty acid synthase) resulting in less hepatic inflammation and steatosis with lower ALT levels compared to mice that were fed ad libitum by high-fat diet of equivalent calories [33].

Sherman et al. [60] showed that time-restricted access to food during the light phase (only 4 hours) with no access to food during the dark phase under 12-hour light/12-hour dark cycle in mice on a high-fat diet prevented obesity, reduced cholesterol levels, and improved insulin resistance compared to mice on high-fat diet ad libitum. In another study, Sherman et al. [59] showed that mice fed only 3 hours during inactivity phase (light phase under 12-hour light/12-hour dark cycle) for 16 weeks had significantly increased amplitude in key metabolic regulators (e.g., peroxisome proliferator-activated receptor *α* mRNA), reduced inflammatory markers (e.g., hepatic interleukin-6 mRNA, tumor necrosis factor-alpha, and nuclear factor *κ*B), and reduced serum cholesterol and triglyceride levels compared to mice fed ad libitum. Average food intake to total body mass ratio in time-restricted and ad libitum feeding groups were almost the same [59]. The results of these three studies suggest that the duration of the food allowance needs to be shorter when the phase of the feeding is changed from dark phase to light phase to produce same metabolic improvement in mice [33, 59, 60]. Indeed, Yasumoto et al. [62] showed that mice fed with high-fat/high-sucrose diet for 8-hours during light phase (instead of 3 to 4 hours in studies as conducted by Sherman et al. [59, 60]) had a significant increase in food consumption, body weight, hepatic triglyceride, cholesterol, and free acid levels as compared with mice fed with high-fat/high-sucrose diet for 8 hours only during the dark phase [62]. That study, by Yasumoto et al. [62], showed that feeding mice with high-fat, high-sucrose diet for long hours during the inactive phase could result in obesity and its complications, at least at short-term [62]. Short duration of this study was a major limitation to draw solid conclusions; it is unknown whether this metabolic dysregulation was secondary to insufficient experiment time provided for murine adaptation to a new feeding pattern. Collectively, the results of these studies suggest that the timing and duration of the food intake may be as important as is the restriction of caloric intake in the prevention of obesity and its metabolic complications [33, 59, 60, 62].

Adamovich et al. [63] showed that wild-type mice fed only at night time had about 50% reduction in hepatic triglyceride levels compared to wild-type mice fed ad libitum. In contrast, clock-disrupted (Per1/2^−/−^) mice fed only at night time had about 25% increase in hepatic triglyceride levels compared to clock-disrupted mice fed ad libitum [63]. Interestingly, they found that the oscillations of about 17% of hepatic lipids had a circadian pattern in both wild-type and clock-disrupted mice [63]. The results of this study suggest that hepatic accumulation of triglycerides is both feeding time- and clock-dependent [63].

Several key regulators of glucose and lipid homeostasis have a circadian rhythm set by light-dark cycles and feeding times [33, 59, 63–65]. The typical, western human diet is generous, often with three daily meals and snacks and, as such, individuals, in essence, remain in a postprandial state throughout the daytime waking hours. We believe that this eating behavior may negate the opportunity for circadian rhythm-mediated weight loss and its effects would correspond to the food-induced obesity and complications observed in mice fed ad libitum [33]. The proposition that strict regulation of feeding times in human could favor decreased hepatic inflammation and sequelae (i.e., cirrhosis, hepatocellular carcinoma) is herein put forth. The idea that postprandial inflammation occurs is supported by the observation that liver stiffness is increased after meals and persists for several hours with little time to recover between meals [66] and that with fasting, and postprandially, serum high-sensitivity C-reactive protein was shown to correlate with plasma triglycerides, chylomicrons, and VLDL after the CHO/fiber but not after a diet rich in monounsaturated fatty acids [67]. This is supported by the observation that liver stiffness in increased after meals and persists for several hours with little time to recover between meals [66].

## 4. Impact of Time-Restricted Feeding on Gut Microbiome in Murine Models

The effect of fasting and feeding patterns on metabolism can be closely associated with alterations in gut microbiota. Zarrinpar et al. [68] and Thaiss et al. [69] have shown in mice that there were cyclical oscillations in the gut microbiome composition based on different feeding and dietary patterns and rhythms (feeding pattern effect on gut microbiome). Thaiss et al. [69] showed that light-time-fed mice exhibited approximately 12 hours of phase shift and enhancement of rhythmicity in the oscillations of bacterial operational taxonomic units compared to dark-phase-fed mice. Subsequently, Paulose et al. [70] demonstrated that *Enterobacter aerogenes*, a habitant of the human gut, showed swarming activity and motility in the culture that corresponded to host circadian clock oscillations and was melatonin dose- and temperature-dependent (melatonin effect on gut microbiome) [70]. Taken altogether, feeding patterns dictate cyclical changes in gut microbiome [68, 69], and these cyclical changes in the gut microbiome are likely generated by either pinealoycte [71–73], enterochromaffin cell-originated [73–76], or food-originated [77, 78] melatonin bound to melatonin-binding sites in the gut bacteria [70]. However, the type and the exact role of melatonin in changing the composition of gut microbiome through feeding pattern remains to be determined. It would be of particular interest to determine because of the potential to explore new interventions.

Several studies have linked obesity and metabolic syndrome to alterations in the gut microbiome [79–89]. The “obese microbiota” has been shown to play a role in the development of NAFLD [79–89]. The gut microbiota determines host adiposity by affecting the transcription of host genes that play a major role in energy consumption and storage as well as extracting energy from indigestible polysaccharides [82]. Zarrinpar et al. [68] showed that mice fed with high-fat diet only for 8 hours during dark phase had significantly higher *β*-diversity, reduced obesity-associated *Lactococcus* species and significantly increased obesity-protective Ruminococcaceae compared to mice fed with high-fat diet ad libitum under 12-hour light/12-hour dark cycle for 8 weeks. If time-restricted feeding can prevent obesity and complications [33] and exerts cyclical changes in gut microbiome [68] in murine models, it remains to be determined whether time-restricted eating in humans can prevent NAFLD and complications by inducing cyclical changes in the composition of the gut microbiome.

## 5. Dawn-to-Sunset Fasting (Ramadan Fasting Model)

Ramadan fasting is a convenient model of *consecutive rhythmic dawn-to-sunset fasting for a month* that has been practiced for over 1400 years “The Noble QUR'AN, Surah Al-Baqarah (Surah 2: Verse 187)”. The fasting without eating or drinking starts at civil dawn after a predawn breakfast and ends at sunset with a dinner. There are several spiritual meanings associated with Ramadan, including teaching self-discipline and healthy survival practices, attributes typically lacking among the obese. It is unclear what biological factors prompt the most favorable changes toward health, but evidence points to stool microbiota as a key factor, possibly working in concert with circadian rhythm by increasing the melatonin expression in the gut. Ramadan fasting is a type of time-restricted feeding model without any calorie restriction, and only lunch and snacks between breakfast and dinner are skipped. In murine models, time-restricted feeding for several days resets (regulates) the phase of hepatic circadian rhythm [52–55]. This thought to optimize the functioning of key metabolic regulator enzymes of glucose and fatty acid metabolism appears to prevent obesity and its complications [33, 59, 60]. During human Ramadan fasting, consecutive dawn-to-sunset fasting is anticipated to produce similar optimization in key hepatic metabolic regulator enzymes, but this has not yet been directly tested. Avoiding the near perpetual fed-state, which is common in the western culture, may be critical in weight loss, drawing the calories from fatty stores and thereby preventing deposition in the liver and preserving the rhythmic secretion of several key hormones including leptin, ghrelin, and adiponectin that regulate hunger. We contend that preserved daytime activity and timing of major food consumption at transition zones of the day (predawn breakfast and postsunset dinner) may be as important as caloric content and composition in the prevention of obesity and its sequela. This approach is in sharp contrast to feeding throughout the nighttime with activity (nightshift) which has been associated with adverse outcomes [90].

When conducting fasting studies, the distinction between dawn and sunrise is crucial because significant metabolic and hormonal changes in humans start at dawn, not at sunrise [91]. The impact of dawn-to-sunset fasting on circadian rhythm may be assessed by measuring melatonin and cortisol levels and the body temperature as surrogate parameters [92–95]. However, it is currently unknown whether melatonin and cortisol levels and body temperature reflect the oscillations from the central clock or peripheral clock; according to a murine study, during time-restricted feeding, uncoupling of peripheral clocks from central clock occurs [52], and a similar uncoupling would be anticipated to occur in humans.

A study conducted on eight healthy subjects who fasted from dawn-to-sunset showed that acrophase (time at which the amplitude of the circadian rhythm peaks) of the rectal temperature on the 25th day of fasting shifted lagging for 2 to 3 hours (delayed) compared to acrophase detected 15 days before the initiation of fasting [94]. In another study conducted in 10 healthy subjects who fasted from dawn-to-sunset, circadian rhythm of oral temperature was phase-shifted during fasting month (measured at the 6th, 15th, and 28th days) compared to baseline (1 week before the initiation of fasting) with significantly decreased oral temperature values during daytime hours and increased values during nighttime hours [95]. Bogdan et al. [93] conducted a study that investigated the effects of dawn-to-sunset fasting on melatonin and cortisol axis in ten healthy subjects and measured blood melatonin and cortisol levels every 4 hours during fasting. The results of that study showed that 24-hour mean melatonin level and amplitude of melatonin rhythm were decreased on the 23rd day of fasting compared to levels measured one week before the fasting month [93]. During dawn-to-sunset fasting, Bogdan et al. [93] also reported a biphasic cortisol level pattern during 24 hours, and al-Hadramy et al. [92] observed similar findings in a single subject on the 15th day of dawn-to-sunset fasting. Collectively, the results of these studies suggest that dawn-to-sunset fasting resulted in phase shifting in master clock oscillations and this is likely related to a change in sleep patterns during Ramadan for adaptation to new mealtimes.

It is unknown whether dawn-to-sunset fasting also results in uncoupling of the hepatic clock from the master clock in SCN [52, 53, 55] and synchronization of peripheral metabolic regulators to feeding/refeeding cycles and energy demands [33, 59, 60]. The uncoupling of the hepatic clock from the master clock in SCN was shown in mice that were exposed to time-restricted feeding [52, 53, 55]. Demonstration of absolute-uncoupling epiphenomena and changes in hepatic clock mRNA expression in humans related to dawn-to-sunset fasting, without liver tissue studies before and after fasting, will be impossible due to invasiveness. However, this concern might not be relevant to effective interventions leading to decreased steatosis. Many studies have shown that significant weight loss occurs during dawn-to-sunset Ramadan [24, 28–32] but the cause is not clear because total daily caloric intake does not appear to be significantly different.

## 6. Impact of Dawn-to Sunset Fasting on Body Mass Index and Lipid Profile

Many studies have shown that significant weight loss occurs during dawn-to-sunset Ramadan fasting (Table 1) [24, 28–32] but the cause is unclear because total daily caloric intake during Ramadan fasting appears to be the same compared to nonfasting status. Several of these studies showed significantly improved lipid profile in healthy subjects and those with coronary artery disease (CAD), cerebrovascular disease (CVS), and metabolic syndrome [24, 26, 27, 29, 31, 96]. A study conducted on 32 healthy adult men who fasted from dawn-to-sunset for one month demonstrated significant improvement in lipid profile despite significantly higher total daily energy intake compared to that from 1 week before the initiation of fasting [24]. The same authors also reported a persistent effect of fasting one month beyond the last day of fasting that suggested a metabolic-memory epiphenomenon [24]. Their findings were in line with the results of murine studies on time-restricted feeding that showed that the timing and duration of the food intake were as important as the caloric content [33, 59, 60].

Lipid profiles were also shown to improve in a study by Temizhan et al. [31] of 52 subjects who fasted from dawn-to-sunset for one month (Table 2). Improvement in lipid profile after a month of dawn-to-sunset fasting was observed not only in healthy subjects but also in subjects with hypertension, diabetes, metabolic syndrome, CAD, and CVD (Table 2) [26, 27, 29]. The results of a prospective observational study conducted on 82 patients with history of CAD, CVD, or metabolic syndrome who fasted for at least 10 hours from dawn-to-sunset showed significant improvement in lipid profile as well as Framingham 10-years coronary heart disease risk score at the end of one month (*P* < 0.001) [29]. Therefore, the evidence for dawn-to-sunset fasting appears to have benefits that affect multiple biologic systems.

There were also studies that showed conflicting findings (Table 2) [25, 28, 32]. Ziaee et al. [32] found significant improvements in fasting plasma glucose levels, weight loss, and reduction in BMI at the 26th day of fasting, but there was no significant improvement in lipid profiles observed. The authors concluded that change in dietary habits and individual reaction to starvation might have played a role [32]. Similarly, Aksungar et al. [25] reported no significant improvement in lipid profile in 40 healthy subjects after one month of dawn-to-sunset fasting compared to 28 healthy controls who did not fast [25]. However, the authors reported that the HDL risk factor (total cholesterol/high-density lipoprotein ratio) was significantly reduced in those who fasted compared to controls [25].

## 7. Impact of Dawn-to-Sunset Fasting on Blood Metabolome

Mathew et al. [97] performed a targeted metabolomic profiling in 11 healthy subjects who fasted from dawn until sunset for one month. That study showed significantly higher phosphatidylcholine concentrations at the 4th week of dawn-to-sunset fasting compared to the first week of dawn-to-sunset fasting [97]. Li et al. [98] showed that reduction in the molar ratio of phosphatidylcholine to phosphatidylethanolamine resulted in impairment of membrane integrity, development of steatohepatitis, and hepatic failure. Puri et al. [99] showed that subjects with NAFLD had reduced total phosphatidylcholine levels. Deficiencies of several enzymes in phosphatidylcholine synthesis were shown to result in obesity and NAFLD [100]. Collectively, these findings suggest that increased phosphatidylcholine levels observed at the 4th week of dawn-to-sunset fasting [97] may play a critical role in the prevention of NAFLD.

## 8. Impact of Dawn-to-Sunset Fasting on Hypertension and Diabetes

For the effects of dawn-to-sunset fasting on hypertension and diabetes, several studies have shown encouraging results. We identified two studies that reported significant improvement in blood pressure in subjects with cardiovascular disease [27, 29]. A gender- and age-matched study showed that subjects with hypertension had a significant reduction in systolic and pulse pressures at the 4th week of dawn-to-sunset fasting compared to that measured one week before fasting month [27]. Similar improvement in systolic blood pressure was reported by Nematy et al. [29].

A well-designed rigorous study conducted on 32 healthy men showed significant improvement in fasting blood glucose levels measured at the 29th day of dawn-to-sunset fasting compared to prefasting levels [24]. One gender- and an age-matched study conducted on 40 type II diabetic subjects and 40 nondiabetic controls showed significant improvement in diabetic patients' fasting blood glucose levels at the 4th week of fasting compared to prefasting levels [26] and another study conducted on 81 healthy students showed similar results [32]. In contrast, a study conducted by Nematy et al. [29] did not show any significant improvement in glucose metabolism parameters at the end of one month of dawn-to-sunset fasting compared to prefasting levels [29]. This is most likely that the authors combined the laboratory measurements that were performed both during the last week of Ramadan fasting and after the end of Ramadan fasting [29].

## 9. Impact of Dawn-to-Sunset Fasting on Inflammatory and Oxidative Stress

Aksungar et al. [25] reported significant improvement in interleukin-6, high-sensitivity C-reactive protein, and homocysteine levels in 40 healthy women and men after one month of dawn-to-sunset fasting [25]. The improvement in interleukin-6, high-sensitivity C-reactive protein, and homocysteine levels was not observed in 40 healthy controls who did not fast [25]. In contrast to Aksungar et al. [25], Nematy et al. [29] did not find significant improvement in high-sensitivity C reactive protein at the end of one month of dawn-to-sunset compared to that from baseline.

Results of two studies performed in normal controls, hypertensive, and diabetic subjects showed significant improvement in oxidative stress parameters including increased blood glutathione and reduced serum malondialdehyde levels at the 4th week of dawn-to-sunset fasting [26, 27]. Remarkably, even six weeks after stopping dawn-to-sunset fasting, glutathione and malondialdehyde levels remained significantly improved in all groups compared to prefasting levels [26, 27]. However, the interrelationship between oxidative stress, inflammation, dysglycemia, and dyslipidemia and the effects of short-term and long-term dawn-to-sunset fasting on these parameters have not been assessed. We did not find any studies that assessed the short-term or long-term effects of dawn-to-sunset fasting on NAFLD.

## 10. Limitations of Previous Studies on Dawn-to-Sunset Fasting and Recommendations for Future Clinical Trials on Dawn-to-Sunset Fasting

Several studies have shown that the dawn-to-sunset Ramadan fasting could be unfavorable for health [101–103], but those results might be due to multiple inconsistencies with the design, conduct, and interpretation of those studies. We believe that a crucial violation in the Ramadan tradition is skipping predawn breakfast that could contribute to a significant daily caloric deficit and would be expected to promote metabolic dysfunction, increased postprandial insulin levels, and fat oxidation [104] and confound results. In most studies that evaluated the impact of Ramadan fasting on BMI, lipid, and other metabolic profiles, information whether subjects had a predawn breakfast was not provided. During Ramadan, due to predawn breakfast, subjects are at postprandial state if blood parameters are measured in the morning. Several studies conducted on Ramadan fasting did not report whether baseline laboratory parameters measured after an overnight fast before the initiation of Ramadan fasting were compared to laboratory parameters measured a couple of hours before the fast was broken at sunset which would be the equivalent of overnight fast. More importantly, the assessment of several key biomarkers of glucose and lipid metabolism and several hormones including cortisol, melatonin, leptin, ghrelin, and adiponectin requires 24-hour blood monitoring with multiple time points as they exhibit circadian rhythm. Measurement of these parameters with only at one or few time points is expected to bias outcomes and can potentially give falsely increased or decreased levels depending on the time they are measured.

Dawn-to-sunset Ramadan fasting starts at dawn and ends at sunset. Therefore, fasters have two major meals a day, breakfast before dawn and dinner after sunset. They can eat ad libitum from sunset until dawn. In general, it is a common practice for Ramadan fasters to work and fast during the daytime, have a dinner, sleep, and wake up one hour before dawn to eat and restart fasting at dawn. Most of the aforementioned studies did not provide sufficient dietary information on meal content, frequency of meals between sunset and dawn, and sleeping times. Furthermore, subjects might be compelled to secretly break the fast and further confound the desired adaptive response. There was no information on the compliance monitoring with fasting in any of the studies. Well-designed clinical trials that will evaluate the impact of dawn-to-sunset fasting on BMI and key metabolic parameters before, during, and after fasting should bring important health maintenance information.

The long-term health benefits of dawn-to-sunset fasting have not yet been appreciated, but the data support the prospect of favorable outcomes. Individuals with certain medical conditions (e.g., diabetes, renal failure) should check with their physicians before they fast [105, 106]. Diabetics who fast for long hours may be at risk for hypo- or hyperglycemia, diabetic ketoacidosis, and dehydration [105, 106]. Individuals in high risk professions (e.g., pilots, surgeons, and crane operators) may want to be off-duty during the first week of fasting if their metabolism is not used to dawn-to-sunset fasting. It has been shown that people who observe Ramadan fasting regain their usual weight after Ramadan [107]. This is likely related to eating ad libitum and remaining in a postprandial state throughout the daytime waking hours after Ramadan for which no fasting intervention would prevent the detrimental health consequences of this type of eating behavior.

## 11. Human Time-Restricted Feeding Models without Calorie Restriction Other than Dawn-to-Sunset Fasting

There are few human fasting models other than dawn-to-sunset Ramadan fasting in which the caloric reduction was not the goal. Moro et al. [108] randomized 34 male subjects who were resistance-trained to time-restricted feeding or normal diet and matched them for caloric intake. While subjects randomized to time-restricted feeding had meals at 1 pm, 4 pm, and 8 pm, those who were in the normal diet group had meals at 8 am, 1 pm, and 8 pm [108]. At the end of 8 weeks of 16 hours of fasting, the time-restricted feeding group had a significant reduction in fat mass, leptin, triglyceride, total testosterone, insulin growth factor-1, and interleukin-1*β* levels and had a significant increase in adiponectin levels compared to the normal diet group [108].

In a study conducted by Halberg et al. [109], eight healthy subjects fasted for 20 hours (from 22 pm until 6 pm the next day) every other day for 15 days. This study was similar to dawn-to-sunset fasting with exception of skipping breakfast at dawn, fasting every other day for two weeks instead of every day for four weeks, and allowance of water intake during fasting [109]. The results of this study showed that time-restricted feeding every other day for 15 days significantly increased insulin-mediated whole-body glucose uptake rate that was consistent with increased insulin sensitivity [109]. In a similar study, Soeters et al. [110] compared time-restricted feeding to standard diet in 8 healthy subjects who first fasted for 20 hours (from 22 pm until 6 pm the next day) every other day for 2 weeks and had standard diet for 2 weeks at least 4 weeks after the time-restricted feeding. Contrary to the study conducted by Halberg et al. [109], this study failed to show improvement in insulin sensitivity and lipid and protein metabolism [110]. Stote et al. [111] conducted randomized crossover study (there were 11 weeks of washout period between two interventions) in 21 healthy subjects to compare metabolic effects of eating 3 meals daily versus 1 meal daily (to be consumed within 4 hours in the evening). The 1 meal daily group has significantly increased systolic and diastolic blood pressure and reduction in body weight and fat mass [111]. In contrast to dawn-to-sunset fasting that provides a balanced distribution of meals at transition zones of the day (predawn breakfast and postsunset dinner), a long period from sunset until dawn, these models either limited food intake to a short period that may result in excessive fullness in participants [108] or skipped breakfast [109–111] that may be detrimental to metabolism. The consequence of skipping breakfast would be expected to promote metabolic dysfunction that can result in increased 24-energy expenditure [104] and confound results of studies on dawn-to-sunset Ramadan fasting.

## 12. Conclusions and Future Trials

We anticipate that consecutive rhythmic dawn-to-sunset fasting might be a cost-effective intervention to prevent obesity, metabolic syndrome, NAFLD, cirrhosis, and hepatocellular carcinoma if definitely shown to be advantageous, and further studies are needed. The challenge to implementing such a fasting routing would be compliance and lifestyle changes to facilitate such a radical intervention. Future studies should not only focus on short-term effects of dawn-to-sunset fasting (e.g., weight loss, improvement in lipid profile) but also on longer lasting effects (e.g., improvement in oxidative stress parameters, inflammation, prediabetes, and activation of DNA repair mechanisms) as well as study the metabolic memory that one can sustain following a period of dawn-to-sunset fasting.

## Figures and Tables

**Table 1:** Impact of dawn-to-sunset fasting for one month on BMI or weight.

Authors	Groups	Number of subjects	Mean age or age range	Population	Reduction in mean BMI (kg/m^2^) or weight (kg)	
					Comparison between initiation and end of fasting^1,2,3,4,5,6^	*P* value
^1^Adlouni et al. [24]	Men	32	25 to 50	Healthy	69.61 to 67.83	<0.01

^2^Temizhan et al. [31]	Women	27	33	Healthy	23.6 to 23.6	0.05
	Men	25			24.3 to 23.0	0.05

^3^Ziaee et al. [32]	Women	39	23	Healthy	21.3 to 20.9	0.002
	Men	41			23.1 to 22.0	0.136

^4^Chaouachi et al. [28]	Men	15	18	Healthy	22.35 to 21.93	<0.01

^5^Nematy et al. [29]	Women (44), men (38)	82	54	CAD, metabolic syndrome or CVD	28.4 to 27.7	<0.001

^6^Norouzy et al. [30]	≤35 years old (women)	51	40	Healthy	24.1 to 23.7	<0.001
	≤35 years old (men)	31			26.4 to 25.9	<0.001
	36–70 years old (women)	31			27.7 to 27.4	<0.001
	36–70 years old (men)	127			26.8 to 26.4	<0.001

^1^Weight was measured 1 week prior to fasting and on the 29th day of fasting. ^2^BMI was measured on the first day of fasting and last day of fasting. ^3^BMI was measured 3 days before fasting and on the 26th day of fasting. ^4^BMI was measured 4 days before fasting and on the 29th day of fasting. ^5^BMI was measured from 7 days prior to fasting to 2 first days of fasting and from the 27th day of fasting to 6 days after one month of fasting. ^6^BMI was measured 1 week prior to fasting and 1 week after one month of fasting.

**Table 2:** Impact of dawn-to-sunset fasting for one month on lipid profile.

Authors	Groups	Number of subjects	Mean age or age range	Population	Mean total cholesterol		Mean triglycerides		Mean high-density lipoprotein (HDL) cholesterol		Mean low-density lipoprotein (LDL) cholesterol		Mean very low-density lipoprotein (VLDL) cholesterol	
					Comparison between initiation and end of fasting^1,2,3,4,5,6,7,8^	*P* value	Comparison between initiation and end of fasting^1,2,3,4,5,6,7,8^	*P* value	Comparison between initiation and end of fasting^1,2,3,4,5,6,7,8^	*P* value	Comparison between initiation and end of fasting^1,2,3,4,5,6,7,8^	*P* value	Comparison between initiation and end of fasting^1,2,3,4,5,6,7,8^	*P* value
^1^Adlouni et al. [24]	Men	32	25 to 50	Healthy	Decreased	<0.001	Decreased	<0.001	Increased	<0.001	Decreased	<0.001	Not reported	

^2^Temizhan et al. [31]	Women	27	33	Healthy	Decreased	0.001	Decreased	0.01	Increased	0.05	Decreased	0.01	Decreased	0.01
	Men	25			Decreased	0.01	Decreased	0.05	Increased	0.05	Decreased	0.05	Decreased	0.05

^3^Ziaee et al. [32]	Women	39	23	Healthy	Increased	0.78	Decreased	0.42	Decreased	0.20	Increased	0.075	Decreased	0.33
	Men	41			Increased	0.51	Increased	0.09	Decreased	<0.001	Increased	0.237	Increased	0.12

^4^Aksungar et al. [25]	Women	20	20 to 39	Healthy	Increased	NS	Decreased	NS	Increased	<0.01	Increased	NS	Not reported	
	Men	20			Decreased	NS	Increased	NS	Increased	NS	Increased	NS		

^5^Chaouachi et al. [28]	Men	15	18	Healthy	Increased	<0.05	Decreased	NS	Increased	<0.01	Increased	<0.05	Not reported	

^6^Nematy et al. [29]	Women (44), men (38)	82	54	CAD, metabolic syndrome, or CVD	Decreased	0.023	Decreased	0.000	Increased	0.000	Decreased	0.000	Decreased	0.000

^7^Al-Shafei [27]	Normal control	40	55	Normal healthy or hypertensive	Decreased	NS	Decreased	S	Increased	NS	Decreased	NS	Not reported	
	Hypertensive	40			Decreased	NS	Decreased	S	Increased	S	Decreased	S		

^8^Al-Shafei [26]	Normal control	40	55	Normal healthy or diabetic	Decreased	NS	Decreased	S	Increased	NS	Decreased	NS	Not reported	
	Diabetic	40			Decreased	NS	Decreased	S	Increased	NS	Decreased	NS		

^1^Lipid profile was measured 1 week prior to fasting and on the 29th day of fasting. ^2^Lipid profile was measured on the first day of fasting and the last day of fasting. ^3^Lipid profile was measured 3 days before fasting and on the 26th day of fasting. ^4^Lipid profile was measured 1 week prior to fasting and at the 4th week of fasting. ^5^Lipid profile was measured 4 days before fasting and on the 29th day of fasting. ^6^Lipid profile was measured from 7 days prior to fasting to 2 first days of fasting and from the 27th day of fasting to 6 days after 1 month of fasting. ^7, 8^Lipid profile was measured 1 week prior to fasting and at the 4th week of fasting. S = statistically significant (*P* < 0.05), NS = not statistically significant; actual *P* values were not reported.

## References

[1] Obesity and overweight. http://www.who.int/mediacentre/factsheets/fs311/en/.

[2] Controlling the global obesity epidemic. http://www.who.int/nutrition/topics/obesity/en/.

[3] Calle E. E., Rodriguez C., Walker-Thurmond K., Thun M. J. (2003). Overweight, obesity, and mortality from cancer in a prospectively studied cohort of U.S. adults. *The New England Journal of Medicine*.

[4] Takuma Y., Nouso K. (2010). Nonalcoholic steatohepatitis-associated hepatocellular carcinoma: our case series and literature review. *World Journal of Gastroenterology*.

[5] Yasui K., Hashimoto E., Komorizono Y. (2011). Characteristics of patients with nonalcoholic steatohepatitis who develop hepatocellular carcinoma. *Clinical Gastroenterology and Hepatology*.

[6] Mittal S., El-Serag H. B., Sada Y. H. (2016). Hepatocellular carcinoma in the absence of cirrhosis in United States veterans is associated with nonalcoholic fatty liver disease. *Clinical Gastroenterology and Hepatology*.

[7] May A. L., Freedman D., Sherry B., Blanck H. M., Centers for Disease Control and Prevention (CDC) (2013). Obesity — United States, 1999–2010. *MMWR Supplements*.

[8] Hammond R. A., Levine R. (2010). The economic impact of obesity in the United States. *Diabetes, Metabolic Syndrome and Obesity: Targets and Therapy*.

[9] Lazo M., Hernaez R., Eberhardt M. S. (2013). Prevalence of nonalcoholic fatty liver disease in the United States: the Third National Health and Nutrition Examination Survey, 1988–1994. *American Journal of Epidemiology*.

[10] Kanwal F., Kramer J. R., Duan Z., Yu X., White D., El-Serag H. B. (2016). Trends in the burden of nonalcoholic fatty liver disease in a United States cohort of veterans. *Clinical Gastroenterology and Hepatology*.

[11] Younossi Z. M., Blissett D., Blissett R. (2016). The economic and clinical burden of nonalcoholic fatty liver disease in the United States and Europe. *Hepatology*.

[12] Ashtari S., Pourhoseingholi M. A., Zali M. R. (2015). Non-alcohol fatty liver disease in Asia: prevention and planning. *World Journal of Hepatology*.

[13] Buzzetti E., Pinzani M., Tsochatzis E. A. (2016). The multiple-hit pathogenesis of non-alcoholic fatty liver disease (NAFLD). *Metabolism*.

[14] Schuppan D., Schattenberg J. M. (2013). Non-alcoholic steatohepatitis: pathogenesis and novel therapeutic approaches. *Journal of Gastroenterology and Hepatology*.

[15] Musso G., Gambino R., Cassader M., Pagano G. (2010). A meta-analysis of randomized trials for the treatment of nonalcoholic fatty liver disease. *Hepatology*.

[16] Chanoine J. P., Hampl S., Jensen C., Boldrin M., Hauptman J. (2005). Effect of orlistat on weight and body composition in obese adolescents: a randomized controlled trial. *JAMA*.

[17] Sanyal A. J., Chalasani N., Kowdley K. V. (2010). Pioglitazone, vitamin E, or placebo for nonalcoholic steatohepatitis. *New England Journal of Medicine*.

[18] Sherman V. (2013). Bariatric surgery. *Texas Heart Institute Journal*.

[19] Weiner R. A. (2010). Surgical treatment of non-alcoholic steatohepatitis and non-alcoholic fatty liver disease. *Digestive Diseases*.

[20] Liou A. P., Paziuk M., Luevano J. M., Machineni S., Turnbaugh P. J., Kaplan L. M. (2013). Conserved shifts in the gut microbiota due to gastric bypass reduce host weight and adiposity. *Science Translational Medicine*.

[21] Rodriguez-Ramiro I., Vauzour D., Minihane A. M. (2016). Polyphenols and non-alcoholic fatty liver disease: impact and mechanisms. *The Proceedings of the Nutrition Society*.

[22] Shi K. Q., Fan Y. C., Liu W. Y., Li L. F., Chen Y. P., Zheng M. H. (2012). Traditional Chinese medicines benefit to nonalcoholic fatty liver disease: a systematic review and meta-analysis. *Molecular Biology Reports*.

[23] Weinstein D. H., Twaddell W. S., Raufman J. P., Philosophe B., Mindikoglu A. L. (2012). SlimQuick™ - associated hepatotoxicity in a woman with alpha-1 antitrypsin heterozygosity. *World Journal of Hepatology*.

[24] Adlouni A., Ghalim N., Benslimane A., Lecerf J. M., Saile R. (1997). Fasting during Ramadan induces a marked increase in high-density lipoprotein cholesterol and decrease in low-density lipoprotein cholesterol. *Annals of Nutrition & Metabolism*.

[25] Aksungar F. B., Eren A., Ure S., Teskin O., Ates G. (2005). Effects of intermittent fasting on serum lipid levels, coagulation status and plasma homocysteine levels. *Annals of Nutrition & Metabolism*.

[26] Al-Shafei A. I. (2014). Ramadan fasting ameliorates oxidative stress and improves glycemic control and lipid profile in diabetic patients. *European Journal of Nutrition*.

[27] Al-Shafei A. I. (2014). Ramadan fasting ameliorates arterial pulse pressure and lipid profile, and alleviates oxidative stress in hypertensive patients. *Blood Pressure*.

[28] Chaouachi A., Chamari K., Roky R. (2008). Lipid profiles of judo athletes during Ramadan. *International Journal of Sports Medicine*.

[29] Nematy M., Alinezhad-Namaghi M., Rashed M. M. (2012). Effects of Ramadan fasting on cardiovascular risk factors: a prospective observational study. *Nutrition Journal*.

[30] Norouzy A., Salehi M., Philippou E. (2013). Effect of fasting in Ramadan on body composition and nutritional intake: a prospective study. *Journal of Human Nutrition and Dietetics*.

[31] Temizhan A., Tandogan I., Dönderici O., Demirbas B. (2000). The effects of Ramadan fasting on blood lipid levels. *The American Journal of Medicine*.

[32] Ziaee V., Razaei M., Ahmadinejad Z. (2006). The changes of metabolic profile and weight during Ramadan fasting. *Singapore Medical Journal*.

[33] Hatori M., Vollmers C., Zarrinpar A. (2012). Time-restricted feeding without reducing caloric intake prevents metabolic diseases in mice fed a high-fat diet. *Cell Metabolism*.

[34] Méndez-Hernández P., Dosamantes-Carrasco L. D., Siani C. (2016). Mealtime habits and risk of developing the metabolic syndrome or insulin resistance among Mexican adults. *The British Journal of Nutrition*.

[35] Goyal A., Terry P. D., Superak H. M. (2014). Melatonin supplementation to treat the metabolic syndrome: a randomized controlled trial. *Diabetology & Metabolic Syndrome*.

[36] Peschke E., Muhlbauer E. (2010). New evidence for a role of melatonin in glucose regulation. *Best Practice & Research Clinical Endocrinology & Metabolism*.

[37] Zanuto R., Siqueira-Filho M. A., Caperuto L. C. (2013). Melatonin improves insulin sensitivity independently of weight loss in old obese rats. *Journal of Pineal Research*.

[38] Sun H., Wang X., Chen J. (2016). Melatonin improves non-alcoholic fatty liver disease via MAPK-JNK/P38 signaling in high-fat-diet-induced obese mice. *Lipids in Health and Disease*.

[39] Zoncu R., Bar-Peled L., Efeyan A., Wang S., Sancak Y., Sabatini D. M. (2011). mTORC1 senses lysosomal amino acids through an inside-out mechanism that requires the vacuolar H^+^-ATPase. *Science*.

[40] Thomson E., Ferreira-Cerca S., Hurt E. (2013). Eukaryotic ribosome biogenesis at a glance. *Journal of Cell Science*.

[41] Cao R., Li A., Cho H. Y., Lee B., Obrietan K. (2010). Mammalian target of rapamycin signaling modulates photic entrainment of the suprachiasmatic circadian clock. *The Journal of Neuroscience*.

[42] Jouffe C., Cretenet G., Symul L. (2013). The circadian clock coordinates ribosome biogenesis. *PLoS Biology*.

[43] Guerrero-Vargas N. N., Navarro-Espíndola R., Guzmán-Ruíz M. A. (2017). Circadian disruption promotes tumor growth by anabolic host metabolism; experimental evidence in a rat model. *BMC Cancer*.

[44] Evans J. A. (2016). Collective timekeeping among cells of the master circadian clock. *The Journal of Endocrinology*.

[45] Reppert S. M., Weaver D. R. (2002). Coordination of circadian timing in mammals. *Nature*.

[46] Gaddameedhi S., Selby C. P., Kaufmann W. K., Smart R. C., Sancar A. (2011). Control of skin cancer by the circadian rhythm. *Proceedings of the National Academy of Sciences of the United States of America*.

[47] Gaddameedhi S., Selby C. P., Kemp M. G., Ye R., Sancar A. (2015). The circadian clock controls sunburn apoptosis and erythema in mouse skin. *The Journal of Investigative Dermatology*.

[48] Sancar A., Lindsey-Boltz L. A., Gaddameedhi S. (2015). Circadian clock, cancer, and chemotherapy. *Biochemistry*.

[49] Bray M. S., Young M. E. (2009). The role of cell-specific circadian clocks in metabolism and disease. *Obesity Reviews*.

[50] Tong X., Yin L. (2013). Circadian rhythms in liver physiology and liver diseases. *Comprehensive Physiology*.

[51] Hatori M., Panda S. (2015). Chapter seven - response of peripheral rhythms to the timing of food intake. *Methods in Enzymology*.

[52] Damiola F., Le Minh N., Preitner N., Kornmann B., Fleury-Olela F., Schibler U. (2000). Restricted feeding uncouples circadian oscillators in peripheral tissues from the central pacemaker in the suprachiasmatic nucleus. *Genes & Development*.

[53] Hara R., Wan K., Wakamatsu H. (2001). Restricted feeding entrains liver clock without participation of the suprachiasmatic nucleus. *Genes to Cells*.

[54] Satoh Y., Kawai H., Kudo N., Kawashima Y., Mitsumoto A. (2006). Time-restricted feeding entrains daily rhythms of energy metabolism in mice. *American Journal of Physiology - Regulatory, Integrative and Comparative Physiology*.

[55] Stokkan K. A., Yamazaki S., Tei H., Sakaki Y., Menaker M. (2001). Entrainment of the circadian clock in the liver by feeding. *Science*.

[56] Yamazaki S., Numano R., Abe M. (2000). Resetting central and peripheral circadian oscillators in transgenic rats. *Science*.

[57] Acuña-Castroviejo D., Reiter R. J., Menéndez-Peláez A., Pablos M. I., Burgos A. (1994). Characterization of high-affinity melatonin binding sites in purified cell nuclei of rat liver. *Journal of Pineal Research*.

[58] Kowalska E., Brown S. A. (2007). Peripheral clocks: keeping up with the master clock. *Cold Spring Harbor Symposia on Quantitative Biology*.

[59] Sherman H., Frumin I., Gutman R. (2011). Long-term restricted feeding alters circadian expression and reduces the level of inflammatory and disease markers. *Journal of Cellular and Molecular Medicine*.

[60] Sherman H., Genzer Y., Cohen R., Chapnik N., Madar Z., Froy O. (2012). Timed high-fat diet resets circadian metabolism and prevents obesity. *The FASEB Journal*.

[61] Zheng B., Larkin D. W., Albrecht U. (1999). The *mPer2* gene encodes a functional component of the mammalian circadian clock. *Nature*.

[62] Yasumoto Y., Hashimoto C., Nakao R. (2016). Short-term feeding at the wrong time is sufficient to desynchronize peripheral clocks and induce obesity with hyperphagia, physical inactivity and metabolic disorders in mice. *Metabolism*.

[63] Adamovich Y., Rousso-Noori L., Zwighaft Z. (2014). Circadian clocks and feeding time regulate the oscillations and levels of hepatic triglycerides. *Cell Metabolism*.

[64] Kentish S. J., Vincent A. D., Kennaway D. J., Wittert G. A., Page A. J. (2016). High-fat diet-induced obesity ablates gastric vagal afferent circadian rhythms. *The Journal of Neuroscience*.

[65] Yu H., Xia F., Lam K. S. (2011). Circadian rhythm of circulating fibroblast growth factor 21 is related to diurnal changes in fatty acids in humans. *Clinical Chemistry*.

[66] Kjærgaard M., Thiele M., Jansen C. (2017). High risk of misinterpreting liver and spleen stiffness using 2D shear-wave and transient elastography after a moderate or high calorie meal. *PLoS One*.

[67] Bozzetto L., De Natale C., Di Capua L. (2013). The association of hs-CRP with fasting and postprandial plasma lipids in patients with type 2 diabetes is disrupted by dietary monounsaturated fatty acids. *Acta Diabetologica*.

[68] Zarrinpar A., Chaix A., Yooseph S., Panda S. (2014). Diet and feeding pattern affect the diurnal dynamics of the gut microbiome. *Cell Metabolism*.

[69] Thaiss C. A., Zeevi D., Levy M. (2014). Transkingdom control of microbiota diurnal oscillations promotes metabolic homeostasis. *Cell*.

[70] Paulose J. K., Wright J. M., Patel A. G., Cassone V. M. (2016). Human gut bacteria are sensitive to melatonin and express endogenous circadian rhythmicity. *PLoS One*.

[71] Lerner A. B., Case J. D., Mori W., Wright M. R. (1959). Melatonin in peripheral nerve. *Nature*.

[72] Reiter R. J. (1991). Pineal melatonin: cell biology of its synthesis and of its physiological interactions. *Endocrine Reviews*.

[73] Bubenik G. A. (2002). Gastrointestinal melatonin: localization, function, and clinical relevance. *Digestive Diseases and Sciences*.

[74] Raikhlin N. T., Kvetnoy I. M. (1976). Melatonin and enterochromaffine cells. *Acta Histochemica*.

[75] Raikhlin N. T., Kvetnoy I. M., Tolkachev V. N. (1975). Melatonin may be synthesised in enterochromaffin cells. *Nature*.

[76] Kvetnoi I. M., Raikhlin N. T., Tolkachev V. N. (1975). Chromatographic detection of melatonin (5-methoxy-N-acetyltryptamine) and its biological precursors in enterochromaffin cells. *Doklady Akademii Nauk SSSR*.

[77] Acuña-Castroviejo D., Escames G., Venegas C. (2014). Extrapineal melatonin: sources, regulation, and potential functions. *Cellular and Molecular Life Sciences*.

[78] Reiter R. J., Rosales-Corral S., Coto-Montes A. (2011). The photoperiod, circadian regulation and chronodisruption: the requisite interplay between the suprachiasmatic nuclei and the pineal and gut melatonin. *Journal of Physiology and Pharmacology*.

[79] Furlow B. (2013). Gut microbe composition and metabolic syndrome. *The Lancet Diabetes & Endocrinology*.

[80] Musso G., Gambino R., Cassader M. (2010). Obesity, diabetes, and gut microbiota: the hygiene hypothesis expanded?. *Diabetes Care*.

[81] Tilg H., Kaser A. (2011). Gut microbiome, obesity, and metabolic dysfunction. *The Journal of Clinical Investigation*.

[82] Tilg H., Moschen A. R., Kaser A. (2009). Obesity and the microbiota. *Gastroenterology*.

[83] Tilg H. (2010). Obesity, metabolic syndrome, and microbiota: multiple interactions. *Journal of Clinical Gastroenterology*.

[84] Bäckhed F., Ding H., Wang T. (2004). The gut microbiota as an environmental factor that regulates fat storage. *Proceedings of the National Academy of Sciences of the United States of America*.

[85] Bäckhed F., Manchester J. K., Semenkovich C. F., Gordon J. I. (2007). Mechanisms underlying the resistance to diet-induced obesity in germ-free mice. *Proceedings of the National Academy of Sciences of the United States of America*.

[86] Le Roy T., Llopis M., Lepage P. (2013). Intestinal microbiota determines development of non-alcoholic fatty liver disease in mice. *Gut*.

[87] Machado M. V., Cortez-Pinto H. (2012). Gut microbiota and nonalcoholic fatty liver disease. *Annals of Hepatology*.

[88] Miura K., Ohnishi H. (2014). Role of gut microbiota and Toll-like receptors in nonalcoholic fatty liver disease. *World Journal of Gastroenterology*.

[89] Devaraj S., Hemarajata P., Versalovic J. (2013). The human gut microbiome and body metabolism: implications for obesity and diabetes. *Clinical Chemistry*.

[90] Qin L. Q., Li J., Wang Y., Wang J., Xu J. Y., Kaneko T. (2003). The effects of nocturnal life on endocrine circadian patterns in healthy adults. *Life Sciences*.

[91] Wehr T. A., Aeschbach D., Duncan W. C. (2001). Evidence for a biological dawn and dusk in the human circadian timing system. *The Journal of Physiology*.

[92] al-Hadramy M. S., Zawawi T. H., Abdelwahab S. M. (1988). Altered cortisol levels in relation to Ramadan. *European Journal of Clinical Nutrition*.

[93] Bogdan A., Bouchareb B., Touitou Y. (2001). Ramadan fasting alters endocrine and neuroendocrine circadian patterns. Meal-time as a synchronizer in humans?. *Life Sciences*.

[94] Roky R., Chapotot F., Hakkou F., Benchekroun M. T., Buguet A. (2001). Sleep during Ramadan intermittent fasting. *Journal of Sleep Research*.

[95] Roky R., Iraki L., HajKhlifa R., Lakhdar Ghazal N., Hakkou F. (2000). Daytime alertness, mood, psychomotor performances, and oral temperature during Ramadan intermittent fasting. *Annals of Nutrition & Metabolism*.

[96] Adlouni A., Ghalim N., Saïle R., Hda N., Parra H. J., Benslimane A. (1998). Beneficial effect on serum apo AI, apo B and Lp AI levels of Ramadan fasting. *Clinica Chimica Acta*.

[97] Mathew S., Krug S., Skurk T. (2014). Metabolomics of Ramadan fasting: an opportunity for the controlled study of physiological responses to food intake. *Journal of Translational Medicine*.

[98] Li Z., Agellon L. B., Allen T. M. (2006). The ratio of phosphatidylcholine to phosphatidylethanolamine influences membrane integrity and steatohepatitis. *Cell Metabolism*.

[99] Puri P., Baillie R. A., Wiest M. M. (2007). A lipidomic analysis of nonalcoholic fatty liver disease. *Hepatology*.

[100] van der Veen J. N., Kennelly J. P., Wan S., Vance J. E., Vance D. E., Jacobs R. L. (2017). The critical role of phosphatidylcholine and phosphatidylethanolamine metabolism in health and disease. *Biochimica et Biophysica Acta (BBA) - Biomembranes*.

[101] Ajabnoor G. M., Bahijri S., Borai A., Abdulkhaliq A. A., Al-Aama J. Y., Chrousos G. P. (2014). Health impact of fasting in Saudi Arabia during Ramadan: association with disturbed circadian rhythm and metabolic and sleeping patterns. *PLoS One*.

[102] Bahammam A. (2006). Does Ramadan fasting affect sleep?. *International Journal of Clinical Practice*.

[103] Bahijri S., Borai A., Ajabnoor G. (2013). Relative metabolic stability, but disrupted circadian cortisol secretion during the fasting month of Ramadan. *PLoS One*.

[104] Nas A., Mirza N., Hägele F. (2017). Impact of breakfast skipping compared with dinner skipping on regulation of energy balance and metabolic risk. *The American Journal of Clinical Nutrition*.

[105] Crosby S. S., Rourke E. J., Warfa M. A. (2005). Fasting and medical issues during Ramadan. *JAMA*.

[106] Sunni M., Brunzell C., Nathan B., Moran A. (2014). Management of diabetes during Ramadan: practical guidelines. *Minnesota Medicine*.

[107] Hajek P., Myers K., Dhanji A. R., West O., McRobbie H. (2012). Weight change during and after Ramadan fasting. *Journal of Public Health*.

[108] Moro T., Tinsley G., Bianco A. (2016). Effects of eight weeks of time-restricted feeding (16/8) on basal metabolism, maximal strength, body composition, inflammation, and cardiovascular risk factors in resistance-trained males. *Journal of Translational Medicine*.

[109] Halberg N., Henriksen M., Söderhamn N. (2005). Effect of intermittent fasting and refeeding on insulin action in healthy men. *Journal of Applied Physiology*.

[110] Soeters M. R., Lammers N. M., Dubbelhuis P. F. (2009). Intermittent fasting does not affect whole-body glucose, lipid, or protein metabolism. *The American Journal of Clinical Nutrition*.

[111] Stote K. S., Baer D. J., Spears K. (2007). A controlled trial of reduced meal frequency without caloric restriction in healthy, normal-weight, middle-aged adults. *The American Journal of Clinical Nutrition*.

